# Instrumentation in Diffuse Optical Imaging

**DOI:** 10.3390/photonics1010009

**Published:** 2013-12-15

**Authors:** Xiaofeng Zhang

**Affiliations:** Department of Radiology, Duke University Medical Center, DUMC 3808, Durham, NC 27710, USA

**Keywords:** diffuse optical imaging, instrumentation, fluorescence, molecular imaging, bioluminescence, tomography, clinical, preclinical

## Abstract

Diffuse optical imaging is highly versatile and has a very broad range of applications in biology and medicine. It covers diffuse optical tomography, fluorescence diffuse optical tomography, bioluminescence, and a number of other new imaging methods. These methods of diffuse optical imaging have diversified instrument configurations but share the same core physical principle – light propagation in highly diffusive media, i.e., the biological tissue. In this review, the author summarizes the latest development in instrumentation and methodology available to diffuse optical imaging in terms of system architecture, light source, photo-detection, spectral separation, signal modulation, and lastly imaging contrast.

## 1. Introduction

Technology development of diffuse optics in biomedicine has been accelerated by the rapid and exciting advances of photonics. There exist a large number of excellent review articles in the literature regarding methodology, mathematical treatment, and applications of the diffuse optics [1, 2, 3, 4, 5, 6, 7, 8, 9, 10, 11]. In this review, the author intends to address this topic from an instrumentation point of view by broadly covering various types of instruments used in diffuse optics with emphasis on biomedical diffuse optical imaging (DOI). Due to the availability of large amount of work on the theoretical bases of DOI methods in the literature, theoretical analysis is not explicitly included and, rather, is distributed along with the relevant instrumentation. The author is also hoping this work offers points of entry for the interested readers to explore the particular subtopics in depth.

The discussions presented in this review article are divided into the following six categories: system architecture, light source, photo-detection, spectral separation, signal modulation, and imaging contrast. Unlike in the other established biomedical imaging modalities, such as magnetic resonance imaging (MRI), x-ray computed tomography (x-ray CT), positron emission tomography (PET), single-photon emission computed tomography (SPECT), and ultrasound imaging, the instrumentation in diffuse optics is highly diversified and can be optimized for the specific needs of the particular applications. As shown in the following sections, the various instruments and methods that are available to diffuse optics can be configured in a number of different ways to accomplish the specific goals. In addition, a particular piece of instrument may be used in different ways and combined with different matching instruments.

## 2. System Architecture

**Figure 1:**
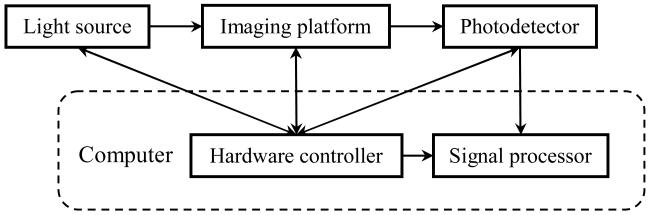
Functional block diagram of a typical DOI system (the arrows show the direction of signal and data flow).

At the system level, a complete DOI setup consists of five functional blocks: the light source, imaging platform, photodetector, hardware controller, and signal processor, shown in Figure 1. Among these functional blocks, only the light source, imaging platform, and photodetector are widely accepted as independent components of instrumentation. The hardware controller functions either as an integral part of a specific piece of hardware or as a part of the computer. The signal processor is mainly the computer with various software including mathematical models, signal processing algorithms, and reconstruction methods. In this review, the term system architecture refers to the relationship of the light source and photodetector with respect to the imaging platform/subject.

### 2.1. Light Coupling

Fiber-coupled illumination and photo-detection have been adopted since the very early stage of development of diffuse optics and are still being widely used. It can be found in diffuse optical tomography (DOT), fluorescence diffuse optical tomography (FDOT), and near infrared spectroscopy (NIRS) of the human brain [12, 13, 14], human breast [15, 16, 17], and small animals [18, 19, 20, 21]. The fiber optics can be in direct contact with the surface of imaging subject or in indirect contact via optical matching fluid. In most applications, multimode fibers are better suited because of their larger numeric aperture that allow higher coupling efficiency, their higher power delivery capability, and their lower system cost compared to the single-mode fibers. Conversely, if maintaining the coherence of light or reducing pulse dispersion is required, using single-mode fibers is the optimal solution [22]. Nonetheless, the specific choice of single-mode or multi-mode fibers optics is also dependent on other factors of the instruments and applications, such as the signal-to-noise ratio (SNR). For example, it was shown very recently that multi-mode fibers significantly improved the SNR in diffuse correlation spectroscopy due to increased admittance of signals [23]. In multispectral imaging, light generated from multiple diverging sources can be simultaneously coupled into a single optical fiber, e.g., using a tilted coupling lens [24]. The advantages of fiber-coupling include high flexibility in hardware configuration and improved instrument ruggedness. Optical fibers are also often used in small animal imaging, human neuroimaging, and multimodality imaging, because they provide reliable optical contact, reduce the interference from hair, and allow flexibility in system configuration and experimental setup, e.g., [14, 25, 26, 27, 28, 29]. Disadvantages of fiber-coupling include limited mobility of the imaging subject, reduced usable experiment duration due to discomfort, and inconsistent skin-fiber coupling efficiency as a result of variations in fiber contact and subject motion.

In noncontact light coupling designs, epi-illumination (aka reflectance) and trans-illumination (aka transmission) configurations are the most common choices. In the epi-illumination configuration, the photodetector is on the same side of the imaging subject as the light source and measures the reflectance; whereas in trans-illumination, the photodetector is on the opposite side and measures the transmittance. Epi-illumination is best suited for imaging superficial features, and can use either unfocused or focused/collimated light source to achieve widefield or point illumination. In particular, widefield epi-illumination is widely used in commercial general-purpose fluorescence imagers for the small animals. In the trans-illumination configuration, a point light source (e.g., a laser beam or focused light spot) is typically used to produce sufficient photon density to allow deep-penetration of the imaging subject. The collimated light can be directed to the imaging subject via scanning devices, such as galvo-scanners or translation stages [30, 31]. This method is best suited for deep-tissue tomographic imaging.

### 2.2. Subject Positioning

In biomedical DOI, the common imaging subjects include parts of human body (e.g., the brain and breast) or whole small animals (e.g., mouse and rat). In human DOI, subject positioning is relatively straightforward and standardized. In animal imaging, on the contrary, subject positioning is much more versatile and affects imaging performance in complicated ways.

The most commonly adopted method for small animal positioning is to use a tablet or flatbed glass to support the imaging subject [32]. Widefield illumination is typically used with this positioning method, but can be either on the same side or on the opposite side of the animal with respect to platform i.e., either epi- or trans-illuminations, respectively. Compared to other configurations, this design is less costly for instrumentation and easier for animal setup, but offers limited depth resolution and reconstruction accuracy in tomographic imaging [33].

Analog to the designs of x-ray CT scanners, the light sources (typically collimated, in a trans-illumination configuration) and photodetectors can be mounted on a gantry that rotates about the imaging subject. This design enables full-angle data acquisition, which results in deep tissue penetration and isotropic image resolution. However, it requires precision manufacturing and motion control of the large and heavy gantry, which significantly increases the system cost and reduces instrument portability. Nonetheless, it can become a highly attractive design for multimodal imaging when integrated with x-ray CT [34, 35].

A simple yet effective alternative way to achieve full-angle acquisition is to use angled or cone-shaped reflective mirrors [36]. This method is especially suited for multimodal imaging, in which the animal must be positioned within a confined space, e.g., the magnet bore of the MRI scanner, the gantry of the x-ray CT scanner, and the scintillator ring of the PET or SPECT scanner. However, some engineering obstacles must be overcome, e.g., variation of focal distance due to the differences in mirror location, geometrical distortions due to the curved mirrors, and development of calibration methods.

A major challenge in diffuse optics is to accurately model photon migration in the irregular-shaped and highly heterogeneous biological tissue, which is computationally costly with existing modeling methods. An alternative solution is to modify the imaging geometry by immersing the imaging subject (e.g., the small animal or human breast) in optical index-matching fluid [37, 38]. As a result of optical index-matching, the boundary conditions for photon migration modeling is transformed from a highly irregular shape to a simple cylinder or slab geometry, which have elegant analytical solutions if the internal inhomogeneity of the imaging subject can be neglected. A variation of this method is to use an imaging cassette that fits tightly around the animal and slightly compress the skin, thereby creating a well-defined and self-contained imaging geometry. An additional advantage of using the imaging cassette is that it can be easily transferred to other imaging equipment (e.g., MRI, x-ray CT, and PET/SPECT) without disturbing the animal posture, allowing accurate and reliable registration of separately acquired data sets.

A tradeoff solution between system complexity and imaging performance is to rotate the animal while maintaining the imaging devices stationary. In this type of setups, the animal is typically positioned and rotated vertically to maintain its posture and minimize interference with optical configuration. This method can be used together with the imaging chamber or individually for free-space full-angle acquisition [30, 39].

## 3. Light Source

### 3.1. Lamp

Lamps are the most commonly used general-purpose light sources because they are simple to use and easy to maintain. With proper choice of bandpass filters, halogen lamps are well suited for epi-illumination fluorescence imaging. A notable limitation of the conventional lamps is that it is difficult to control and calibrate the output optical power accurately, making quantitative measurement difficult without real-time monitoring of the optical power. Using regulated power supply, variations in the output optical power can be mitigated, e.g., MH100 by Dolan-Jenner (Boxborough, MA).

The most commonly used lamps are metal-halide lamps and mercury arc lamps, which operate at high temperatures and produce strong UV and infrared components in the spectrum, making them difficult and costly to use in imaging. Additional equipment is usually required to remove the heat, ozone, and nitrogen oxide generated by the lamps due to the high temperature and UV emission. Because they typically require 30 min to reach stable operation after ignition and 30 min to properly cool down before re-ignition, the lamps often must be kept on between imaging sessions or experiments, which can significantly reduce their usable lifespan.

### 3.2. Laser

Diode lasers have always been the primary choices for collimated illumination in diffuse optics. Because of their compact size, rugged structure, low power consumption, and long lifespan, the laser diodes (LDs) are highly attractive candidates for system integration, particularly in portable, handheld, and/or battery-operated devices. Above a threshold current, the output optical power of the LDs are linearly related to the driving current intensity under a given operating temperature, which makes them well suited for quantitative measurement. In frequency domain (amplitude modulated) and time domain (pulsed) measurements, the LDs are almost exclusively used because they are easy to modulate and can be quickly switched on and off to generate a pulse train or to achieve time-multiplexed acquisition. Interested readers are referred to the topical reviews on biomedical applications of diode lasers [40].

LDs have little or limited wavelength tunability. For applications that require wavelengths varying over a broad range, the best choice is typically tunable titanium-doped sapphire (Ti:Sapphire) lasers. The Ti:Sapphire lasers are solid-state lasers that have a continuous tuning range typically between 680 and 1080 nm. It produces a very narrow (~100 fs) and fast (~80 MHz) laser pulse train with an average output optical power of >1 W at 800 nm. With the recent advances in photonics, hands-free and maintenance-free commercial Ti:Sapphire laser systems are becoming widely available. The leading choices of hands-free pulsed Ti:Sapphire lasers are the Chameleon product series by Coherent and the Mai Tai series by Spectra-Physics (both located at Santa Clara, CA). The main drawbacks of the Ti:Sapphire lasers include large physical dimensions, high energy consumption, high system cost, and laser safety concerns.

In recent years, supercontinuum lasers have clearly become an attractive alternative to the Ti:Sapphire lasers and are increasingly gaining attention because of their broad emission spectrum (typically 400-2500 nm with a time-averaged power spectral density of ~1 mW/nm in the visible-NIR range at 20 MHz repetition rate) and picosecond-pulse generation (<100 ps), e.g., the WhiteLase series by Fianium (Southampton, United Kingdom) and the SuperK series by NKT Photonics (Birkerod, Denmark). With the proper filters, a supercontinuum laser can be used as a universal tunable pulsed laser source. Note that the main difference between a Ti:Sapphire laser and a supercontinuum laser is that the former produces a monochromatic laser at a given time and can be tuned to specified wavelengths, whereas the latter produces a broadband “white-light” laser. In diffuse optics, the effective pulse width is limited by the highly scattering biological tissue; and the allowable optical power is limited to ~10 mW for collimated beams. Note that the exact maximum allowable power (MAP) is dependent on wavelength, spot size, pulse width, repetition rate, and type of tissue (refer to the ANSI Z136 Standards).

The features under investigation in DOI are often deeply embedded in the tissue, e.g., fluorescently labeled tumors or functionally activated cortical areas of the brain. As a result, the temporal point-spread function (TPSF) of the imaging subject in deep-tissue DOI is significantly broader than that of the laser pulse. In addition, the lifetime constants of commonly used organic fluorophores in diffuse optics are on the order of 0.5-5 ns, making picosecond-lasers sufficient for time domain sampling. For these reasons, ultrafast femtosecond-lasers are not only unnecessary, but may also be detrimental in typical setups of diffuse optics as they emit significantly higher instantaneous power that limits the usable average optical power due to laser safety concerns.

### 3.3. Light Emitting Diode

With recent advances in photonics, light-emitting diodes (LEDs) are replacing many of the light sources that have been conventionally fulfilled by lamps and even lasers. One of the most notable advances is the availability of high-power LEDs that can output optical power comparable to that of the conventional halogen lamps. Compared to the conventional lamps, another attractive feature of the LEDs is that they can be modulated in both the frequency and time domains because of their excellent output linearity, short rise/fall time (1-10 ns), and low driving current (0.1-1 A). Among the commercially available LEDs for diffuse optics, the modulation bandwidth is currently on the order of 100-1000 MHz, and the minimum pulse width is typically 2-20 ns. Despite the fundamental differences in coherence and collimation between the LDs and LEDs, the performance of these two technologies that are pertinent to diffuse optics are becoming increasingly comparable in terms of output optical power, spectral width, and pulse width. In addition, the LED light sources are generally safer than the lasers, which often makes LEDs the preferred choices in handheld devices, e.g., [41]. In a recent report that compared the SNRs of fluorescence in capillary gel electrophoresis, LEDs and lasers produced similar results despite small variations in baseline and stability [42]. It is worth noting that the LEDs and LDs share some similarities but are distinct in important features, as summarized in Table 1.

## 4. Photo-detection

### 4.1. Discrete Photo-sensitive Element

Photomultiplier tubes (PMTs) are well-established highly sensitive photo-detection devices. When photons impinge on the photocathode, an electron is generated and subsequently streamed toward a series of cascaded dynodes by a focusing electrode. Photo-multiplication is achieved via multi-stage acceleration of the electrons along the cascaded dynodes by an electric field. The exiting electrons that are collected by the anode form an amplified signal of electric current. PMTs are widely used in fiber-based configurations, particularly when the number of photodetectors are not very large (typically less than ~100). PMTs have high sensitivity, high gain, short rise time, and excellent linearity, but are limited by their complex circuitry (requiring fast-switching of the high-voltage electric field), bulky size, and noticeable aging effect.

Avalanche photo-diodes (APDs) are relatively new members in the family of photodetectors for diffuse optics [43]. The APDs are also termed as the “solid-state PMTs” because they are semiconductor devices that achieve optical signal amplification in a way similar to that of the PMTs: incoming photons are eventually converted to electric current signals via avalanche breakdown within the reverse biased *p-n* junctions of the semiconducting materials. Although some key parameters of the APDs are not as impressive as the PMTs (in particular, higher dark current and lower signal gain), the APDs have shown high potentials because of their compact size, highly integrated circuitry, low power consumption, and low voltage operation (20-100 V for APDs vs. ~1000 V for PMTs).

A special type of the APDs is the single-photon avalanche diode (SPAD). Contrary to the APDs, the SPADs are biased above the avalanche voltage. Upon photon impact, a massive avalanche breakdown current is generated to achieve single-photon detection sensitivity. As a result, the SPADs are best suited for photon-counting rather than analogue photo-electric signal amplification.

The conventional photo-sensing methods convert the influx of photons to electric signals (current or voltage). Under the low-light conditions, the signal gain and/or integration time must be increased to reach sufficient SNR for signal readout. In ultralow-light imaging, however, this approach becomes insufficient because the signal intensity may become comparable to the noise due to the dark current and the readout process of the photo-sensing device. A less-utilized but highly sensitive method, termed time-correlated single-photon counting (TCSPC), e.g., SPC-150 by Becker & Hickl and PicoHarp 300 by PicoQuant (both at Berlin, Germany), can be used to overcome this limitation. TCSPC measures the photon statistics by time-tagging the individual incoming photons [44]. A TCSPC system consists of an ultrafast photodetector and a photon-counter. When a photon impinges on the photodetector, which can be a PMT or a SPAD, it generates a photoelectron that is subsequently amplified by orders of magnitude and produces an electric pulse. The timing of this electric pulse is recorded by the photon-counter with respect to the timing of the pulsed laser source. Eventually, the characteristics of the optical signal is revealed by the distribution of the photon time-tags. This method is particularly well suited for ultralow light measurements because it has single-photon sensitivity and is not affected by the dark current and readout noise. Furthermore, the performance of the TCSPC method is mainly determined by the timing precision of the photon counter rather than the TPSF of the front-end photodetector, making it an attractive solution for applications that require high sensitivity, less pulse broadening, and true signal representation. A number of noncontact small animal DOI systems using the TCSPC method with PMTs have been developed in recent years [45, 46, 47].

### 4.2. Integrated Photo-sensing Array

Many applications of diffuse optics require a large number of photodetectors, particularly when high spatial resolution is desired. The primary choice to meet this need is indisputably the charge-coupled devices (CCDs). Optical emission from the imaging subject can be coupled to the CCD either via an imaging lens or fiber optics. Using CCDs, high-density spatial sampling can be achieved easily, which dramatically mitigates the limitation of the underdetermined inverse problem in diffuse optical reconstruction [37]. A CCD is an array of *p-*doped metal-oxide-semiconductor capacitors integrated with charge-transfer readout circuits. Photons impinged at these biased metal-oxide interface are converted into electrons and are subsequently stored in the capacitors. By the end of the imaging integration time, the stored electric charges are transferred to the readout registers and quantized via analog-to-digital conversion. Variations in photonic designs will result in CCD chips that have different characteristics in photon-electron conversion efficiency, dark current generation, image transfer, and readout noise, each of which is a compounded result of multiple factors in the photonic designs. There exists a large number of manufacturers of scientific CCD cameras. The specific choice of camera is dependent on the specifications of the imaging experiment, e.g., signal intensity, image matrix size, and frame rate.

Designed for low-light imaging, an intensified CCD (ICCD) is basically an image intensifier coupled to a CCD. The image intensifier is mainly consisting of a micro-channel plate (MCP) that is located between a photocathode and a phosphor screen. The photocathode converts the incoming photons into photoelectrons, which are accelerated toward the MCP by a high-voltage electric field between the two components. The MCP is a 2D array of continuous-dynode electron multiplier (i.e., the micro-channels) that amplifies the input photoelectrons with a gain controlled by an adjustable bias voltage. The amplified electron beam exits the MCP and impinges on the phosphor screen and is converted to photons, which are coupled to the CCD for signal sensing. Coupling of the optical output from the image intensifier to the CCD can be implemented either with lenses, e.g., PicoStar HR by LaVision (Goettingen, Germany) or fiber optics, e.g., PI-MAX 4 by Princeton Instruments (Trenten, NJ). The lens-coupling configuration is simple and detachable, thereby doubling the ICCD camera as a CCD camera. However, this configuration suffers from low coupling efficiency and are subject to lens-related optical aberrations. In contrast, fiber-coupling gives significantly higher coupling efficiency but typically has honey cone-shaped artifact in the images when the light level is low. The image intensifier often also functions as an ultrafast electronic shutter in time-resolved acquisition. Although the typical use of the ICCDs is in time domain photo-sensing, they can also been used in the frequency domain by modulating the MCP bias voltage with RF signals [48].

Electron multiplying CCD (EMCCD) is essentially a CCD with on-chip electron multiplication and charge-to-voltage conversion stages to significantly improve the SNR by reducing the contribution of image readout noise, e.g., iXon series by Andor Technology (Belfast, United Kingdom). Compared to the conventional CCDs, the EMCCDs offer high performance in low-light imaging in terms of short integration time and high SNR thanks to the electron multiplication stage. In particularly, the frame rate in low-light imaging is significantly improved. Despite their higher costs, EMCCDs have become highly attractive upgrade replacement in many applications that conventionally use CCDs, e.g., [49].

Complementary metal-oxide semiconductor (CMOS) imaging sensors were invented in the same era as the CCDs, but are only recently available for scientific imaging – therefore often termed sCMOS to emphasize that their performance meets scientific-grade specifications, e.g., the ORCA-Flash series by Hamamatsu Photonics (Shizuoka, Japan). Different from the CCDs, which output analog signals of the image pixel-by-pixel, the CMOS imagers produce digitized signals of all pixels in parallel. As a result, although the CMOS imagers suffer from lower optical efficiency due to reduced pixel filling factor, they are better suited for applications that require high frame rate and larger dynamic range [50, 51].

In diffuse optics, the most relevant specification is arguably the SNR, which is a collective result of the imaging instruments, operational parameters, and experimental conditions. The signal level is given by the photon fluence rate multiplied by the integration time and the photo-electric amplifications, whereas the noise level is a complex function of the quantum noise, dark current noise, readout noise, amplifier noise figure, and digitization noise. A rule of thumb for continuous wave imaging is that under moderate-to-high light conditions, the CCD is the best performer; whereas under ultralow-light conditions, the EMCCD consistently outperforms the ICCD, which is in turn superior to the CCD. Nonetheless, in practice, the specific choice on the imaging sensor has to be made based on the particular application and imaging equipment. For example, the quantum noise can become dominance in imaging as the CCD can use back-illumination (on back-thinned chips) to significantly improve the quantum efficiency from typically 50% to >90% in the visible spectrum, whereas the photon admittance rate of the image intensifier in the ICCD can be considerably degraded in order to accomplish ultrafast gating.

## 5. Spectral Separation

In fluorescence and multispectral imaging, optical filters are routinely used for spectral separation. The most commonly used are dielectric interference filters: band-pass/-stop and long-/short-pass edge filters that are used to selectively admit or reject certain band of the light spectrum. Depending on the specific optical design, dichroic filters may also be used to separate mixed optical signals that share the same light path. Because interference filters discriminate different wavelengths of light through the thickness of their dielectric coatings, varying the light incident angle can significantly alter the center wavelength of this type of filters. This is a factor that must be taken into consideration in designing lens-coupled photo-detection instruments because the viewing angle of the imaging lens can be fairly large in biomedical imaging. For example, assuming a typical viewing angle of 45° for the imaging lens, the admitted fluorescent emission contains a substantial component that is 26 nm shorter than the specified center wavelength of 800 nm with normal light incident, using the formula given in [52] and assuming the effective index of refraction of the fused silica substrate is 1.5. As a result, partial overlapping of the excitation and emission bands may occur and introduce considerable amount of background signal and significantly reduces imaging contrast and detection sensitivity. On the other hand, this angle-dependent shift of the center wavelength has been successfully used to produce tunable filters that can achieve a tuning range of 10-20% of the center wavelength at normal incidence by varying the incident angle.

Other spectral separation methods that are available to diffuse optics include standard spectrometers that are based on refraction prism or diffraction grating, liquid crystal tunable filters [53], and acousto-optic tunable filers [54]. With the continuously tunable filters, multi-fluorophore discrimination can be dramatically enhanced by spectral deconvolution [55]; and high-content fluorescent samples can be quickly and effectively analyzed using synchronous fluorescence, which scans the spectrum of interest with fixed excitation-emission wavelength distance [56].

## 6. Signal Modulation

### 6.1. Amplitude Modulation

Frequency domain measurement methods are established on amplitude modulation of the excitation light and demodulation of the detected optical emission. Amplitude modulation can be conveniently applied to the LDs and LEDs by modulating the driving current (within the linear range) with sinusoidal waves. Mathematically speaking, the driving current of LDs and LEDs can be expressed as *S* = *DC* + *AC* × *sin*(*ωt* + φ), where *S* is the modulated signal, *DC* is the time-averaged signal, intensity, *AC* is the modulation intensity, *ω* is the angular modulation frequency, *φ* is the modulation phase delay, and *t* is time. Note that the variables *DC* and *AC* represent the terms borrowed from the electrical terminology of direct current (DC) and alternating current (AC), which are the two independent frequency components of the driving current in amplitude modulation. The *DC* signal (i.e., the signal offset) is predominantly determined by the absorption coefficient of tissue; the *AC* signal (i.e., the modulation) is determined jointly by the absorption and scattering coefficients; and the phase signal *φ* reflects the mean time of flight of the photons through the tissue, a function of the tissue scattering coefficient. By increasing the modulation frequency, the SNR of the phase and modulation depth (defined as *AC/DC*) measurements can be significantly improved [57].

**Figure 2:**
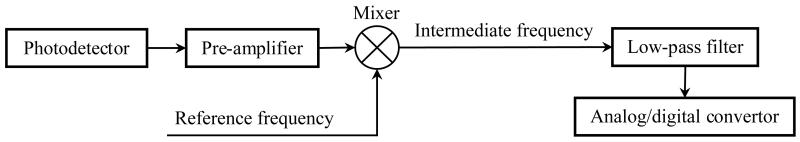
Generalize diagram of the heterodyne and homodyne demodulation architectures.

Among the reported circuitry architectures for signal demodulation in frequency domain diffuse optics, heterodyne and homodyne are most commonly adopted because of their high efficiency and simplicity. The heterodyne and homodyne architectures share the same strategy in radiofrequency (RF) signal detection in that the input high-frequency RF signal is multiplied by a reference signal in an analog mixer and down-shifted to a lower frequency for sampling, Figure 2. This detection strategy significantly simplifies circuit design and improves performance in demodulation because of the shift from high-frequency to low-frequency. The main difference between these two architectures is the source of the reference frequency: the heterodyne architecture uses a high-precision local oscillator (LO) as the reference and generates an intermediate frequency (IF, also known as the beat frequency) signal that is the difference of the RF and the LO frequency; and by contrast, the homodyne architecture uses the carrier wave as the reference signal and directly transfer the RF signals to the baseband, and therefore is also known as the Zero IF or Direct-Conversion architecture. Generally speaking, the heterodyne architecture has better sampling performance in terms of precision and noise, and the homodyne architecture is significantly simpler and less expensive to implement.

The frequency domain instruments typically use LDs or LEDs as the light sources and use PMTs or APDs as the photodetectors. The source and detectors are usually fiber-coupled and adopt a topology to cover the imaging area with multiple source-detector distances. A particular source-detector combination defines a measurement channel. These measurement channels can be either time-multiplexed or frequency-multiplexed. In the time-multiplex design, only one source is activated at a time while all the detector are receiving. The sources are activated and deactivated in turn using RF switching (positive-intrinsic-negative diodes, resonance selection circuits, or relay) or optical switches [58, 59, 60]. In the frequency-multiplexed design, heterodyne circuitry architecture must be used to produce IF signals at different frequencies for frequency-based separation at the signal sampling stage. It is noteworthy that the strategy of frequency-multiplexing can also be applied to multichannel continuous-wave instruments with very low modulation frequency to improve the efficiency of acquisition [61].

An important subcategory of the frequency domain measurement methods is phase detection, which is highly sensitive to the changes in tissue scattering coefficient [62, 63]. Quadrature detection (i.e., separating the real and imaginary parts of the RF signal) and zero-cross detection are the two main methods of choice. Note that amplitude-phase crosstalk exists due to the changes in detector rise time in response to the varying light intensity, and due to internal RF coupling of the detector, particularly when the modulation depth is low [64]. Using the zero-cross method, a highly compact handheld device for breast cancer screening was developed, which identifies potential inhomogeneity in breast tissue by detecting the phase difference [41]. A comprehensive review on instrumentation for signal phase detection using the frequency domain method is given in [63].

### 6.2. Temporal Modulation

**Figure 3:**
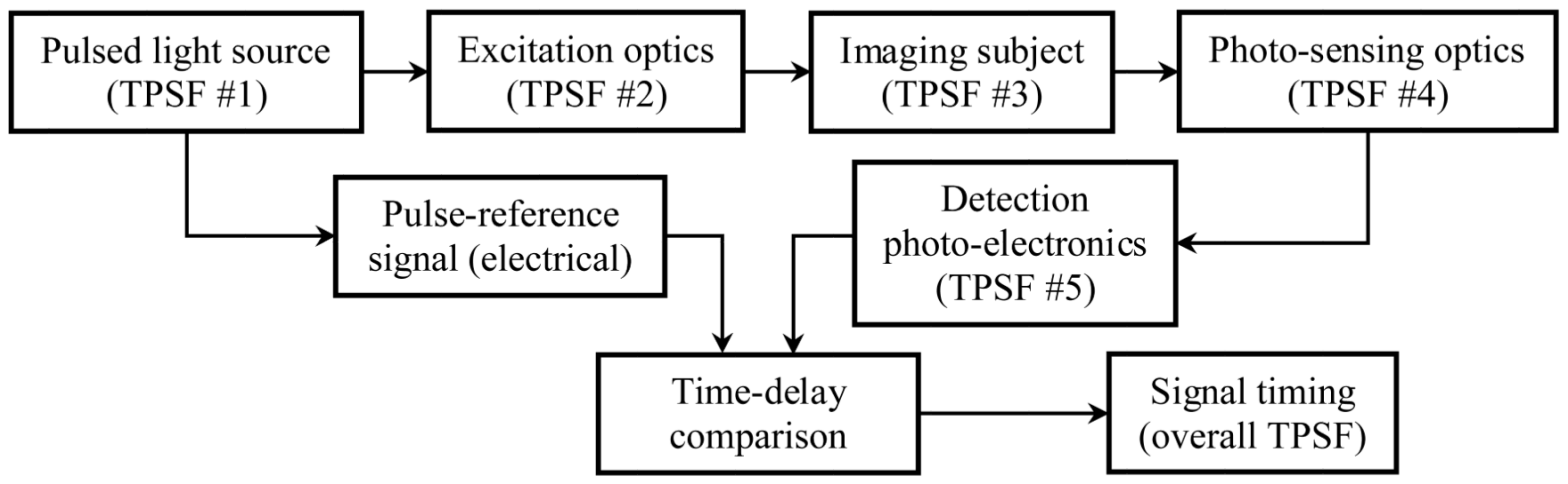
Schematics of the temporal modulation methods.

Time domain methods in diffuse optics are primarily established on time-resolved measurement of the light emission from biological tissues in response to pulsed optical excitation, Figure 3. Lasers have been most frequently used in this area of applications because of their ability to generate stable pulse train with narrow pulse width and high optical power density. Recently, high-performance pulsed LEDs are available commercially and are finding increasingly more applications in this area because of their simplicity and low cost. Using time-resolved photo-sensing methods, the spread of the temporally focused (i.e., narrow pulse) light input is characterized to reveal the underlying biological tissue: the pulse intensity signifies tissue attenuation, the pulse width is tightly related to tissue scattering, and the pulse delay is related to both tissue thickness and scattering. More in-depth discussions of the quantitative relation of these parameters can be found in [62, 65, 66, 67, 68, 69, 70]. A special case of the temporal modulation methods is the so-called early photon (also known as ballistic photon) imaging method, which uses ultrafast laser and detector time-gating to reduce the TPSF of the imaging system, thereby improving the spatial resolution [71, 72, 73].

From a system point of view, the acquired signal is the product of the input light pulse convolved with the transfer functions of the excitation optics, the biological system under imaging, the photo-sensing optics, and the photo-electronics for signal detection. Mathematical analysis and experimental verifications in the time domain have been conducted extensively for diffuse optics in the past, e.g., as described in [62, 65, 66, 74, 75, 76, 77, 78]. As a rule of thumb, the overall TPSF of the diffuse optics system can be approximated by summing the characteristic time delays and pulse widths of the TPSFs of the constituent subsystems. It is widely accepted that the temporal resolution in time domain DOI is limited by the photodetector or the imaging subject, for a representative system with the following specifications: the lasers has a pulse width of 100 fs to 10 ps full width at half maximum (FWHM); the intensifier gating speed of the ICCD cameras is 200 ps or greater [79]; and the TPSF of biological tissue typically has a FWHM on the order of nanosecond dependent on the tissue thickness and the scattering coefficient [74].

### 6.3. Spatial Modulation

Spatial domain modulation is a relatively new method in DOI. It uses spatially modulated light source to create structured illumination with adjustable patterns that effectively samples at multiple points in the space-frequency domain [80, 81, 82]. In terms of instrumentation, spatial modulation can be achieved in a number of different ways, e.g., by using glass templates [83], micro-mirror arrays [80], liquid crystal devices [84, 85], or potentially other devices, e.g., [86, 87]. With the recent advances in semiconductor technology, spatial modulation in DOI is primarily implemented using 2D micro-mirror arrays in integrated circuit chips that can be conveniently controlled directly via digital signals or in the form of digital projectors [80]. This type of devices are commonly referred to as digital micro-mirror devices (DMDs), which are essentially chip-level arrays of addressable light switches: the mirrors are mounted on individual microscopic hinges and can be independently tilted toward binary angles (i.e., the “on” and “off” states), creating structured light by selectively reflecting a uniform light source [88]. The spatial modulation method allows deeper penetration compared to the conventional widefield imaging methods [89, 90] and offers higher spatial resolution or faster acquisition speed compared to the laser-scanning methods [91, 92].

### 6.4. Fourier Analysis

The Fourier analysis method is one of the most important tools in signal processing. It is well known that Fourier transform relates the time domain to the time-frequency domain and the spatial domain to the space-frequency domain. As a result, despite the distinct differences in instrumentation, the Fourier method can be used to conveniently process data between the duo of time domain and time-frequency domain [70, 93] as well as between the spatial domain and space-frequency domain [94]. A special application of the Fourier method is the so-called lock-in method, which, by synchronizing the timing of photo-detection to that of the light pulse train, creates an equivalent narrow-band bandpass filter in the time-frequency domain to dramatically improve the signal-to-background contrast [95]. Analog to that in the time-frequency domain, the lock-in method can also be used in the space-frequency domain to augment the contrast of spatially modulated signals [82].

## 7. Imaging Contrast

### 7.1. Tissue Absorption and Scattering

In diffuse optical tomography (DOT) and near infrared spectroscopy (NIRS), the contrast is tissue absorption and scattering coefficients, which alter the intensity, phase/time delay, and spatial/temporal pulse spread of the input light, but not the emission wavelength. In the near infrared spectrum, which spans approximately 650-900 nm, tissue absorption primarily stems from the hemoglobin molecules in the red blood cells. Hemoglobin is an excellent endogenous contrast agent for investigating blood oxygenation and tissue perfusion. Using two wavelengths (e.g., 690 and 830 nm), typically on both sides of the isosbestic point of oxy-hemoglobin and deoxy-hemoglobin at ~800 nm, the concentrations of oxy- and deoxy-hemoglobin can be measured [96], the ratio of which is the level of blood oxygenation, and the sum of which is the level of tissue perfusion. The concentrations of water and lipids can also be investigated with additional photon wavelengths or modulation frequencies [97, 98]. These important physiological parameters have been extensively used in studies of cerebral hemodynamics [14, 28, 60, 63, 90], tumor biology [8, 11, 15, 32, 45, 72, 99, 100, 101, 102, 103], and other diseases [104, 105, 106, 107, 108, 109, 110, 111]. Tissue scattering coefficient has been associated to electrical activity of activated neurons in *in vitro* studies [112, 113, 114]. The changes in scattering coefficient of bulk brain tissue have been observed in the human brain upon functional activation [115, 116].

### 7.2. Fluorescence

Fluorescence diffuse optical tomography (FDOT), also known as fluorescence molecular tomography and fluorescence-mediated tomography (FMT), is an optical equivalent of the established radionuclide molecular imaging. For this reason, it is often considered an important optical molecular imaging method. It visualizes fluorescently labeled features or probes with high sensitivity and, more importantly, without using radioactive tracers. In its most basic applications, the contrast in FDOT is fluorescence intensity, which is linearly related to the fluorophore concentration but is also strongly modulated by its depth in tissue as well as the tissue optical properties. Other characteristics of fluorophores have also been used as the contrast mechanisms, particularly the fluorescence lifetime constant that has been investigated using both frequency domain or time domain methods [44, 69, 102, 117, 118, 119, 120, 121, 122]. With the proper fluorescent probes, FDOT can be potentially used to indirectly visualize a broad range of physiological parameters, such as Ca^2+^ activity [123], neurovascular coupling [124], tissue oxygenation [125], tissue *pH* values [126], cell membrane potential [127], nanomedicine delivery [128], and a number of other parameters that are summarized in [129].

Recently, up-conversion luminescent nanoparticles were introduced into the area of biomedical optical imaging. The defining feature of these up-conversion nanoparticles is that they absorb 2 photons with lower energy (e.g., in the NIR region) and emit at a higher energy level (e.g., red) via a nonlinear conversion process [130]. Compared to the conventional fluorescent probes, this type of nanoparticles have the advantages of deep penetration (due to low-energy excitation photons) and low background signal (far separation of the emission from the excitation band) [131].

### 7.3. Bioluminescence

Bioluminescence is a special imaging method because it does not involve an excitation light source. It is included in this review because its optical emission is within the spectrum of interest (the visible-NIR range), and also because it has similar instrumentation and reconstruction methodology as the DOT and FDOT [132, 133, 134]. A distinct benefit of using bioluminescence is that imaging contrast and sensitivity are orders of magnitude higher than those of FDOT because the background signal induced by the light source is eliminated.

### 7.4. High-energy Radiation

X-ray luminescence is a new addition to the field of DOI. It uses collimated x-ray beam to excite phosphor nanoparticles that emit optical photons in the red-NIR range and reconstruct 3D distributions of the phosphor using the characteristics of the x-ray beam as priors [135, 136, 137]. Recently, the cone beam x-ray source was successfully applied to the same imaging principle, and has significantly improved the imaging throughput [138]. This imaging method has high potentials in biomedical imaging as it inherently combines the high resolution of x-ray CT and the high sensitivity of optical detection.

Cherenkov luminescence occurs when charged particles traveling in a dielectric medium has a velocity greater than that of light in the medium [139]. The significance of this physical phenomenon for biomedical imaging is that visible photons can be observed upon high-energy radiation on biological tissue. In water, the threshold energy of Cherenkov luminescence is 0.263 MeV for electrons [140], making optical detection of *β*-emitting radioactive tracers feasible. This technology is becoming an alternative to the conventional PET imaging in both preclinical and clinical applications [141, 142, 143, 144, 145]. Note that as Cherenkov emission is a broadband spectrum dominated by UV and extending into the NIR region with increasingly lower intensity, deep-tissue imaging is challenging with low concentration of radionuclides and/or short imaging integration time.

A very recent advance in Cherenkov imaging is to use external high-energy radiation sources, such as the linear accelerators, to induce Cherenkov luminescence in tissue [146, 147]. Because the medical accelerators have high radiation energy (e.g., 6-18 MV for x-ray photon beams and 6-21 for electron beams) and dose rate (e.g., 1-200 MU/min), penetration and SNR of Cherenkov imaging are significantly improved. As a result, a number of new applications become feasible, such as Cherenkov-excited fluorescence [148], Cherenkov tissue spectroscopy [149], radiation beam profiling [150], and radiation dose monitoring [151].

## 8. Summary

As shown in this review article, the instruments and methods available for biomedical diffuse optical imaging is highly diversified and flexible. The optimal implementation of the instrumentation is dependent on the given biomedical application: a particular design of instrument outperforms other designs only under specific imaging requirements (e.g., epi-illumination vs. trans-illumination); and similarly, one type of devices can only be best suited for the given experimental conditions (e.g., CCD vs. ICCD). On one hand, it is apparent that designing one best instrument for all applications is an unrealistic task. On the other hand, it is such diversity and flexibility of instrumentation that makes diffuse optics highly adaptive and broadly applicable to so many applications in biology and medicine.

## Figures and Tables

**Table 1:** Comparisons of LED and LD light sources.

	LED	LD
**Technology**	Semiconductor	Semiconductor
**Photo-emission mechanism**	Electroluminescence	Stimulated emission
**Optical power density**	Low	High
**Power consumption**	Low	High
**Power efficiency**	High	Low
**Optical output linearity**	Linear	Linear above threshold
**Temperature stability**	High	Low
**Spectral width**	Broad (~50-100nm)	Narrow (~1-10 nm)
**Wavelength choices**	Less	More
**Wavelength tunability**	No	Yes (limited)
**Directionality**	None	High
**Coherence**	No	Yes
**Polarization**	No	Yes
**Speckling effect**	No	Yes
**Mode of operation**	Multimode	Single- or multi-mode
**Modulation bandwidth**	Low	High
**Lifespan**	Longer	Long
**Cost**	Low	High
**Operation**	Easy/simple	Difficult/complex
